# Report of the Proceedings of the Medical Association of Georgia, for 1861

**Published:** 1861-05

**Authors:** A. G. Thomas

**Affiliations:** Secretary Medical Association of Ga.


### ARTICLE II.

### *Report of the Proceedings of the Medical Association of Georgia, for* 1861.

### FIRST DAY.

The Medical Association of Georgia, met pursuant to adjournment, in the city of Atlanta, at the Atheneum, on the 10th of April, 1861, 10 o'clock, A. M. The President called the house to order. The deliberations were opened with prayer by Rev. Dr. A. Means. On calling the roll the following regular members reported themselves:

Drs. J. F. Alexander, W. V. Aderhold, H. W. Brown, Hayden Coe, T. S. Denny, D. O. C. Heery, Jos. P. Logan, W. P. Harden, D. C. O'Keefe, A. M. Parker, Thomas S. Powell, E. J. Roach, J. N. Simmons, W. A. Shelby, A. G. Thomas, T. C. H. Wilson, J. G. Westmoreland, J. M. Boring, R. J. Massey, J. L. Hamilton, Atlanta; A. M. Boyd, Cave Spring; T. M. Brantly, Meriwether county; W. P. Bond, Lithonia; B. B. Brown, G. A. Black, J. R. McAfee, Dalton; G. G. Crawford, Jonesboro; W. D. Cunningham, Jasper; L. S. Cunningham, Forsyth county; S. H. Dean, Conyers; F. O. Dannelly, Greenville; D. G. Hunt, Calhoun; Alexander Means, Oxford; T. K. Mitchell, A. J. Schaffer, Lawrenceville; W. C. Smith, Grantville; V. H. Taliaferro, Columbus; A. S. Whitaker, Jonesboro; Z. L. Waters, Calhoun; J. N. Coe, Flat Rock; W. C. Brandon, Floyd county.

The minutes of the last meeting were then read.

The following physicians, being suitably vouched for, were unanimously elected members of the Association:

Wm. J. Russell, Lawrenceville; John Baxley, Yellow River; R. N. Payne, J. W. Bailey, C. A. Jones, H. L. Wilson, D. H. Connally, W. C. Moore, Atlanta; A. S. Mayson, Decatur; J. H. Malone, Calhoun; W. H. Mitchell, Griffin; J. I. Harris, Greenville; J. R. Russell, A. S. Fowler, —— Calhoun, Ringgold; E. H. Raushenburg, Varnell's Station; —— Quinn, Red Clay; C. C. Hammond, F. A. Thomas, E. W. Allen, C. P. Gordon, Dalton; J. A. McKown, Snapping Shoals; T. J. Foster, Buchanan.

Of these, Drs. Baxley, Wilson, Mayson, Moore, Harris, Payne and Bailey, were present.

The President having ordered a ballot for officers for the ensuing year, a ballot was taken, which resulted in the election of

Dr. J. T. Banks, of Griffin, President.

Dr. J. F. Alexander, of Atlanta, 1st Vice-President.

Dr. V. H. Taliaferro, of Columbus, 2d Vice-President.

Dr. A. G. Thomas, of Atlanta, Secretary, &c.

On motion of Dr. Means, Editors who were present were invited to seats.

The President appointed Drs. O'Keefe and Taliaferro to induct the President elect into the chair.

On retiring from the chair, Dr. Coe delivered a very interesting and instructive address, enriched with many gems from the classic history of Medicine.

Dr. Banks, on assuming the chair to which he had just been elected, expressed his gratitude to the Association in a few appropriate and happy remarks.

It being stated that both the Orator and Alternate who were elected at last meeting, were absent, on motion of Dr. Logan, the President appointed, as committee to secure the services of a substitute to fill that position, Drs. Logan, Dannelly and Taliaferro.

On motion of Dr. O'Keefe, the Association tendered its thanks to Dr. Coe for the able address he had just delivered before the body, and requested a copy of it for publication.

Dr. Logan presented the following resolution, which was adopted:

*Resolved*, That a committee of five be appointed by the President of this body, to take into consideration the future relations of the Medical Association of Georgia with the American Medical Association, and report at their earliest convenience during this session of this body.

President appointed as that Committee, Drs. Logan, Alexander, Boyd, McAfee and Bond.

On motion, Association adjourned till 3 o'clock, P. M.

AFTERNOON SESSION, 3 o'clock, P. M.

Minutes of forenoon read.

Reports from auxiliary societies being called for, Dr. B. B. Brown reported the formation of a society of physicians residing in the counties of Catoosa, Whitfield and Murray, called the State Line Medical Association, composed of fifteen members, holds monthly meetings, has adopted the Constitution and Code of Ethics of the Medical Association of Georgia, and desired to be admitted as an auxiliary to that body.

On motion of Dr. Coe, the society was admitted as auxiliary.

No report on correspondence. No report from members appointed to present written communications.

The committee appointed to secure an Orator as substitute, through its chairman, Dr. Logan, reported "Dr. A. Means will deliver the Annual Address to-morrow morning at 11 o'clock."

On motion of Dr. Coe, received and adopted.

Voluntary Contributions were called for.

Dr. V. H. Taliaferro presented a very elaborate and interesting report on Typhoid Fever. Dr. F. O. Dannelly presented a report on cases of Typhoid Fever, and one on Snake Bites, with specimens of a plant which is an antidote for the bite of the Rattlesnake, which he presented to the Medical Garden of Atlanta Medical College.

Dr. J. G. Westmoreland, on the part of the Faculty, returned the thanks of their body for the specimen, and presented an invitation to the Association to visit the Medical College; and Dr. A. G. Thomas, on behalf of the Board, invited the body to visit the Atlanta Female Institute, which invitations were accepted.

On motion of Dr. Boyd, adjourned till to-morrow morning, 10 o'clock.

---

### SECOND DAY.

MORNING SESSION, April 11th, 10 o'clock, A. M.

House called to order by the President.

Minutes of yesterday read.

Regular business being resumed, Dr. Smith presented a report of a case of Ovarian Dropsy. Dr. Shaffer presented a splint for fracture of femur, of his own invention; also, an original and attractive instrument, which he calls the Intra Uterine Scoop. This latter instrument elicited the commendation of the whole body.

The hour for the delivery of the Annual Address having arrived, Association adjourned till 2 o'clock, P. M.

At 11 o'clock, Dr. A. Means proceeded to deliver the Annual Address, which was well prepared, well delivered and a very learned production.

---

AFTERNOON SESSION, 2 o'clock, P. M.

House called to order. President in the chair.

Oral communications were called for.

Dr. Banks presented a beautiful specimen of Hydatid Uteri, with a few remarks on the pathology of the disease, requesting the views of the members of the Association on the question, "are Hydatids of this class always the result of conception?" Drs. Dannelly, J. G. Westmoreland, Coe, Powell, and others, expressed their views at some length, which views tended mainly to answer the query in the affirmative. Some of the gentlemen stated, however, that *organized* membranes were sometimes discharged from the virgin uterus, but no certain evidence that such membranes belong to the class under consideration. Dr. Shaffer reported several cases of rupture of membranes and discharge of uterine fluids, a length of time previous to parturition.

Dr. Logan, chairman of committee on relations of this Association with the American Medical Association, reported as follows:

That while this Association acknowledges no abatement of its zeal for the advancing intelligence and success of the profession—to whose interests it is devoted—and contemplates no abandonment of the high code of ethics and the conventional courtesies which have so long governed and distinguished the ranks of regular medicine; yet, the circumstances by which we are now surrounded, not only authorizes, but requires the disruption of long existing ties as indispensable to the maintenance of harmonious action and the continued progress of the great principles to whose destiny we are pledged; therefore,

1. *Resolved*, In the opinion of your committee, the great political revolution which has sundered the *National* ties that have bound us as a part of a Confederate Government of Independent States for three-fourths of a century and spread deep disaffection far and wide through the two sectional divisions of the late "Union," constitute ample and sufficient cause—such as will be sanctioned at the impartial bar of the scientific and professional world—for the prompt and entire disruption of the bonds by which we have been heretofore united to the American Medical Association, and we hereby recommend that they be forthwith *dissolved*.

2. *Resolved*, That whatever may be our grievances as a people—and we hereby declare them to be deep, and in their results upon us abiding—we suffer them not to *control* us in this decision, but declare it to be the calm and deliberate action of those who are desirous to receive the highest moral and scientific results contemplated by this Association.

3. *Resolved*, That we hold ourselves in readiness, as the organized representatives of the Medical Profession of the great State of Georgia, to unite with our sister States of the Confederate States of America, in the formation of a new professional organization for the *South*, upon the same broad and generous principles which we have been ever disposed to honor and maintain, and which shall still continue to meet our warm approval and hearty concurrence.

4. *Resolved*, That in accordance with the foregoing preamble and resolutions, this Association will be no longer represented in the American Medical Association, and hereby declare *its complete and final separation from that body.*

On motion of Dr. Dannelly the report was received and unanimously adopted by a standing vote.

Dr. Powell moved that a committee be appointed to send copies of our resolutions, with regard to the American Medical Association, to the Medical Associations in other States of the Confederate States, and request their co-operation in the formation of a Medical Association of those States. Dr. J. G. Westmoreland offered to amend, by substituting the words "Secretary of the Association," instead of "Committee." Dr. O'Keefe offered as a substitute, for the whole, the following resolution, which was adopted :

*Resolved*, That the secretary communicate the secession action of this body to the various State medical organizations within the limits of the Confederate States, and invite their attention thereto; and also to consider the propriety of organizing a Southern Medical Association.

Dr. J. G. Westmoreland, from committee appointed at last meeting to memorialize legislature, made a report, which, on motion of Dr. Coe, was received, and committee continued till next meeting.

Dr. Logan moved to defer action on the resolution with regard to permanent location of this Association till next meeting. After some discussion the motion was carried.

The place chosen for holding the next meeting was Columbus.

On motion of Dr. Alexander, committee on revision of Constitution and By-Laws was continued, to report at next meeting.

On motion, committee on Medical Literature was discharged.

On motion of Dr. Simmons, committee on Finance was continued till next meeting.

Dr. O'Keefe moved that a committee of three be appointed to raise $100, to be distributed as prizes for the three best Essays presented to next meeting—$50 for the first, $30 for the second, and $20 for the third. Carried. Committee, Drs. O'Keefe, Taliaferro and Boyd.

On motion, the President appointed, as the committee to examine Prize Essays, Drs. H. Coe, A. Means, J. F. Alexander, H. W. Brown and T. C. H. Wilson.

On motion of Dr. Coe, a committee of three was appointed to take convenient time and procure the services of Orator and Alternate for next meeting. Committee, Drs. Coe, Dannelly and Brown.

Dr. O'Keefe offered the following resolution, which was adopted:

*Resolved*, That this Association having been informed upon reliable authority that Dr. W. N. King, of the city of Savannah, has engaged in the irregular practice of medicine (called Homœopathy), therefore, that the Secretary of this body be directed to strike from the roll of this Association the name of Dr. King.

Dr. Dannelly offered the following resolution, which was unanimously adopted:

*Resolved*, That a committee of three be appointed by the chair, to return the thanks of the Association to Dr. Means, for the able, eloquent and interesting address delivered this day, and solicit a copy for publication in one of the medical Journals.

Committee—Drs. Dannelly, Alexander and O'Keefe.

Dr. Denny offered the following resolution, which, on motion of Dr. Logan, was adopted:

*Resolved*, As the sense of this Association, that a system of regular and continuous registration of births, marriages and deaths, is one of the highest importance to the State, to the profession, and to the citizens; and, that this Association, through its officers, as the most influential and general way of advancing the object, hereby declare the necessity of the profession uniting in calling the notice of the Legislature, at its next session, to the great value of a system of registration of births, marriages, and deaths, and that the Association will take prompt and earnest action in bringing prominently before the general assembly the following, as the more important objects attached to the whole subject, viz:

1. To ascertain the relative number and proportion of births, deaths and marriages, in order to ascertain the progress and increase of the population.

2. The causes of deaths, so as to be able to trace the operations of natural and physical causes on the health of tne inhabitants.

3. The season of the year, and duration of illness, and locality where certain diseases prevail, so as to suggest means for abating them.

4. The age, sex, condition, color, nativity, and occupation, in order to know how these various circumstances influence marriages, births and deaths.

5. With a proper and complete record of the events in life are involved great public and private rights, such as claims to property, &c., &c.

Dr. Boyd offered the following resolution, which was adopted:

*Resolved*, That the thanks of this body be tendered to Mr. Williams, the proprietor of the Atheneum, for his generosity in allowing us its use gratuitously.

Dr. O'Keefe offered the following, which, on motion of Dr. Logan, was adopted:

*Resolved*, That the committee on Prize Essays be instructed, in awarding prizes, to give preference to subjects of practical interest to the profession, and to award the prizes publicly, during the session of the Association, in cash or its equivalent, at the discretion of the committee.

Dr. Taliaferro offered the following resolution, which was adopted:

*Resolved*, That the thanks of this Association be tendered to the Medical Fraternity of Atlanta, and its citizens generally, for their kind and hospitable attention to the members of this Association; and, also to those Rail Road Companies who have passed the members to and from this meeting for one fare.

On motion, the President was required to appoint at his convenience a committee of arrangements.

Minutes having been confirmed, the Association then adjourned to meet on the 2d Wednesday in April, 1862, in Columbus.

A. G. THOMAS,
Secretary Medical Association of Ga.

